# A National Early Intervention System as a Strategy to Promote Inclusion and Academic Achievement in Portugal

**DOI:** 10.3389/fpsyg.2017.01137

**Published:** 2017-07-05

**Authors:** Vitor Franco, Madalena Melo, Graça Santos, Ana Apolónio, Leonor Amaral

**Affiliations:** ^1^Department of Psychology, University of ÉvoraÉvora, Portugal; ^2^Research Center for Education and Psychology, University of ÉvoraÉvora, Portugal; ^3^Interdisciplinary Center for History, Culture and Societies, University of ÉvoraÉvora, Portugal; ^4^Associação Portuguesa de Pais e Amigos do Cidadão Deficiente MentalÉvora, Portugal; ^5^ISPA-Instituto UniversitárioLisbon, Portugal

**Keywords:** Early Intervention, inclusion, school achievement, developmental psychopathology, social network

## Abstract

Early intervention with children at risk or facing developmental problems is a practice defined by three fundamental characteristics: being family-centered, being based on the community and on the child’s life context, and being conducted by a team with transdisciplinary practice. In this paper we wish to present how the SNIPI-National System of Early Intervention, implemented in Portugal over the past 15 years, contributes to promote maximum development and the full inclusion of children up to 6 years of age and works to prevent school failure. The SNIPI covers the entire territory and intends to respond to the needs of children with developmental disorders or those in at risk situations. This community-based early intervention model is linked to the health, education and social care systems, involving the three responsible Ministries. In the present community case study, we present the implementation of this program in the Alentejo region, involving 31 local teams and almost 2500 children. Through the regional structure’s reports and the responses of parents and professionals in impact studies, we demonstrate how the system is established and how it tackles school failure and improves the educational inclusion of these children. The impact of this Early Intervention model has been significant not only on children’s developmental outcomes, but also for the health, education and social care professionals who work in a transdisciplinary perspective, as well as for the families who became more skilled at evaluating the children’s needs and the support provided. This approach to implementing a family-centered Early Intervention program can contribute to full inclusion. It facilitates the transition to schooling based on a non-discriminatory approach and educational achievement by aiding development and an adapted contextualization in pre-school education. This program system introduces significant innovation within the framework of existing educational policies that promote development and inclusion, and has therefore earned the interest of the scientific community and policy-makers alike. It has been possible to implement some of principles already studied but it had never been tested. The Early Intervention program in Alentejo, as part of the SNIPI, can be an example of good practices, with its own characteristics that allowed to create a network of integrated and comprehensive responses to the needs of the population in this region.

## Introduction: A Community Based Model of Early Intervention in Portugal

### Historical Perspective

In recent years, Portugal has implemented a National System for Early Intervention (SNIPI) has been implemented. This system covers the entire territory and is intended to respond to the needs of the children with disabilities or at serious risk of developmental delay.

This Early Intervention community based model has, as its first fundamental and innovative feature, the articulation between health, education and social security services, and it is supported by a specific legal framework (Decreto-Lei 281/2009, 2009) resultant from the work of three Ministries.

Usually, care for children with developmental disorders is distributed between three institutions: The Ministry of Social Security is responsible for rehabilitation institutions and organizations working with people with disabilities; The Ministry of Health oversees therapeutic and clinical interventions; and the Ministry of Education safeguards children’s educational needs. Each of these fields has its own methodologies and emphases and all tended to work separately and without major articulation in the past.

The care of children with developmental disorders has progressed in Portugal, as in other countries, since the 1970s with the emergence of expert centers and non-governmental associations. These tried to address the needs of specific groups of children or people with disabilities or some other issues such as intellectual disabilities, cerebral palsy, etc. Later, more alternatives emerged (catering to the children under the age of six) and some of them reflected an effort to combine different fields, in particular health, education and social security.

Prior to the aforementioned regulatory legislation, a dispatch issued by the Government in 1999 (Despacho Conjunto 891/99, 1999) supported more or less the responses already in place throughout the country. These alternatives included the traditional approaches with regards to rehabilitation and reduction of problematics hinging on isolated measures: therapy and special education. All of these interventions focused on the problem and on the child.

The publication of these regulations constituted a fundamental landmark in Early Intervention in Portugal. This new legislation has determined the implementation of a national system, within the framework of a privileged partnership between the three subscribing Ministries, Education, Health and Labor and Solidarity, with the involvement of the NGO’s, legally defined as support entities of the Local Intervention Teams (LIT).

The legal framework of these teams was enacted through the conclusion of cooperation agreements. The concerted action of all these entities gave body to and framed the constitution of multidisciplinary teams. The aim was to provide direct support to children and their families in the context of their daily lives and with the involvement of the community.

For the first time, a type of organization and methodology was defined: A community-centered workgroup based on the capabilities and functioning of the different contexts of the child’s life, and a family-centered intervention methodology.

This organizational system met some implementation difficulties at a national level, but has since matured in some regions where it was possible to create the network of teams that were able to test the feasibility and effectiveness of a nation-wide project.

After a lengthy review process, new legislation (Decreto-Lei 281/2009, 2009) created the National System of Early Intervention (SNIPI) and provided the tools for its implementation throughout the national territory. This legislation preserved the principles of the previous law and reinforced the role of health services in early detection and referral of children with developmental disorders or at risk of serious delay.

### The Structure of the National Early Intervention System (SNIPI)

At the present, the National Early Intervention System is in place across the country, with some small discrepancies in the coverage and even in the operation mode. The SNIPI has a central structure, which is national and which articulates the general policies of operations, involving representatives of the three Ministries involved. There are intermediate structures (regional level) that ensure the implementation of these policies on the ground and provide coordination and supervision of local team’s operations.

At the base of this pyramid, we find LIT which are composed of professionals from different fields. They provide the interventions with families and children, and the coordination with other services and institutions. Some fundamental structural principles are assumed as the basis for professional training and the production of guidelines (Carvalho et al., 2016). Three fundamental premises are emphasized: working with a family centered perspective, this model has to be based on the community and on the child’s contexts, and the interventions have to be conducted by a transdisciplinary team (Guralnick, 2005).

The local teams and the system itself, have as guiding aims for the interventions, to promote the maximum development and also the maximum inclusion of children up to the age of 6. When, at this age, children enter in Basic schooling (which is always done in mainstream schools) is expected that those whose development is altered or threatened will have better success because of the work already developed by early intervention.

## Early Intervention and Prevention of Educational Failure

The entry into compulsory Basic education represents an important challenge, for any child and family, one sometimes experienced with contradictory feelings. Research has shown that the process of transition to formal schooling implies the involvement of the educational agents (i.e., families, teachers, staff) and the interaction between the various organizational contexts of the mesosystem (Bronfenbrenner and Morris, 2006) in order to enable a smooth transition that encourages the development of the child and their sense of belonging to school (O’Kane, 2016).

In the case of children with developmental problems, issues related to the transition to formal education systems (kindergarten, school) are particularly important (Barnes-Holmes et al., 2013), especially regarding school readiness (UNICEF, 2012). But school readiness does not concern simply the child; it involves three interrelated dimensions: (a) ready children; (b) ready schools; and (c) ready families. “Children, schools and families are considered ready when they have gained the competencies and skills required to interface with other dimensions and support smooth transitions” (UNICEF, 2012, p. 3).

As systematically and clearly demonstrated by research, inclusive educational settings give children with special educational needs (SENs) greater academic and social gains.

There are several educational transitions throughout the child’s life, beginning with the passage from the family context (home) to the nursery or kindergarten (including changes of setting in the preschool context: micro-transitions). But the transition to the school context represents the most important educational transition in the child’s life (O’Kane, 2015). Many studies show that positive transition experiences are predictors of better social, emotional, and educational outcomes (Peters, 2010; Sayers et al., 2012; O’Kane, 2015).

The transition to school of children with SENs is particularly stressful for families (Peters, 2010; Barnes-Holmes et al., 2013), in particular due to concerns toward non-continuity of specialized support.

In the case of the national Early Intervention system, these concerns also seem to make sense. Indeed, this system promotes the mutual collaboration between families and technicians for the integral development of the child. By age 6, the child is integrated in a system that is family-centered and community-based.

The Early Intervention teams prepare the transition to preschool and their path in the kindergarten is followed attentively in close cooperation with the families in order to meet any potential emergent needs of the children.

In most cases, SNIPI teams and families are actively engaged in the child’s process of transition of the child to preschool, planning and establishing effective communication with kindergartens and clarifying the roles of the various educational agents, which is a practice that promotes the development of the child and his educational and social success (Peters, 2010; Burgon and Walker, 2013; Ministry of Education, 2014).

The entry into primary school, however, implies more profound changes, both in the expectations of the child’s roles (Bronfenbrenner, 1979) and in the demands of the context itself. Firstly, because the focus is more now on the child and less on the family, as well as on an individual educational plan. But secondly, because entry into formal schooling usually implies a change in child support services; they are now under the responsibility of special educational services (Ministry of Education), which represents a fundamental change regarding the philosophy of Early Intervention approaches.

These changes are accompanied by major concerns of the families, not only regarding the school readiness of their children (UNICEF, 2012), but particularly with regards to potential social relationships in which they may be involved. The issue of child social relationships appears to be one of the main concerns of the families related with their children’s entrance to school (Burgon and Walker, 2013; Ministry of Education, 2013).

Early intervention contributes meaningfully to positive educational transitions, emphasizing collaborative work between all participants involved in the development of the child, reducing risk factors for the child (or increasing the child’s ability to cope with these risk factors), and promoting their mental health – an essential condition to increase the welfare, the possibilities of educational success, and the healthy development of children with SENs (Dunst, 2002; Hirst et al., 2011).

## Community Case Study: Early Intervention Network in Alentejo

### The Early Intervention System in Alentejo

In order to highlight the impact of the Early Intervention System, we can look at how this network has been established in one of the first regions that successfully implemented this system.

Alentejo is the largest region of Portugal occupying one third of the national territory, including most of the south of the country. It consists of 3 districts, and part of another, for a total of 47 counties. The average population density is 23, 4 inhabitants per Km^2^, the lowest in the country and one of the lowest in Europe.

The settlement is mostly concentrated around the district capitals (Évora, Beja and Portalegre) with a significant part of the rural population living in geographical and social isolation. High percentages of aging populations and low school enrollment rates are both present.

In 2015, there were 189 residents aged 65 and over for every 100 under 15 years, which is considerably higher than the national averages.

In Alentejo, the current organization of Early Intervention services began in 2001 with the constitution of the regional and district coordination teams and an initial survey of needs, which led to the construction of a network of county-based teams, including a few isolated teams already working in disability-linked institutions.

In 2002 there were 15 teams, supporting a total of 605 children with developmental problems and, in the years to follow, the number of children supported by Early Childhood Intervention teams significantly increased as this network spread throughout the region. Nowadays, there are 31 LIT that support the needs of children and families in Alentejo, according with the actual legislation (Decreto-Lei 281/2009, 2009), that enforces the national system.

### The Current Early Intervention Network

The current network is organized in a decentralized structure, with three levels of geographic organization depending on a Subcommission of Regional Coordination, which responds to the Commission of National Coordination and assures the management of the necessary resources for the implementation of the system in the region, in accordance with a national plan of action. The LIT have a multidisciplinary makeup. Each team has childhood educators, psychologists, social workers, physical, occupational and speech therapists, and sometimes doctors and nurses, in a total of nearly 300 professionals.

The system also includes 29 NGO’s that ensure the legal framework for the teams through the celebration of Cooperation Agreements with the three responsible Ministries.

In 2015, 2471 children under the age of 6 years were supported in Alentejo (SNIPI-National System of Early Intervention, 2016): 60% of which had a developmental delay without known etiology, 18% were diagnosed with a specific condition or disability, and in 22% cases some development risk factors were identified.

The current model of early childhood intervention requires that the support be provided in the child’s natural setting, with the active participation of parents and principal caregivers, in a partnership based on a trusting and close relationship. During 2015, 14% of the children received support exclusively at home, 37% in the educational setting (kindergarten) and 41% benefited from the support in a mixed setting, that is, at home and at the kindergarten. In 8% of cases it was necessary to resort to another context of intervention, such as primary healthcare facilities and other specialized centers (SNIPI-National System of Early Intervention, 2016).

### Main Concepts and Framework

The evolution in the structure of the SNIPI was accompanied by a conceptual modification. Early Intervention is defined as a measure of integrative support the aim of which is to develop specific actions of a preventive nature in the field of education; health and social security for children from 0 to 6 years, with disabilities, developmental delay or with serious risk of developing handicaps, and their families (Decreto-Lei 281/2009, 2009).

This care system should take into account a perspective of intervention in children’s natural settings of life and to actively involve the principal caregivers, as developmental enhancers, in order to promote social inclusion.

The underlying Early Intervention concept, which has changed over time and according to the prevailing paradigms relating to child support and disability, relies on three fundamental pillars.

Firstly, Early Intervention recognizes the contributions of the neurosciences and the advances of knowledge of brain functions, including the cerebral plasticity (Johnston et al., 2001) as the ability of the brain to remodel according the experiences of life. The malleability and the fast maturation of the central nervous system of the newborn allows acting on possible injuries or disorders, in order to prevent future follow-ups, but it also means that, in situations of risk, the harmful stimuli have a greater impact in the development than in older children, due to the same plasticity. This can lead to a timely intervention (Shonkoff, 2010; Shonkoff and Levitt, 2010).

The knowledge resulting from the studies about child development, enhances the importance of the early times of life for the establishment of future skills, with a special emphasis on the mother–infant relationship and attachment as a secure base from which the child explores the world and acquires developmental skills. The transactional approach to development (Sameroff, 1983, 2010; Sameroff and Mackenzie, 2003) has been especially important in the field of early intervention. Research has equally shown children’s precocious abilities to establish relationships to be crucial to their development. This perspective justifies an intervention focused on the family.

Bioecological contributions and perspectives are equally important (Bronfenbrenner, 1979; Bronfenbrenner and Morris, 2006) and enhance the systemic importance of life contexts in the promotion of development. Hence the support should be geared at strengthening the abilities needed for the child to reach autonomy and social inclusion (Guralnick, 2008, 2011).

SNIPI constructs its theoretical framework on the evidence-based research (Shonkoff, 2010) and presents itself as an intervention framework that whilst representing an investment, is a more effective application of resources with a improved development quality, thereby avoiding higher costs in the future (Guralnick and Conlon, 2007).

### Innovative Aspects of the Program

This regional program presents innovative aspects which have led to a successful outcomes, more so than in other regions of the country, where the implementation has been much more difficult. The recognition that the needs of children with development disorders can only be fully evaluated, interpreted and tackled within the family and social setting the development takes place, implies community actions and with them the involvement of all local and broader resources throughout the region. Proximity solutions allowed a significant reduction of the children and families transport costs, which were almost entirely supported by the health system.

Furthermore, this program allowed a reduction in social and economic costs for families, given the presence of professionals in their home settings. At present, teams have cars, acquired by the Regional Health Administration of Alentejo, with community co-financing resources, which facilitates professional visits, and children and family travel, mainly in situations of great geographic isolation.

Building strong partnerships enabled creating adequate responses to the needs of children and families, with the involvement of the regional administrations of health services, education and social security. The articulation of these services has further developed the program and the monitoring of responses in order to reduce imbalances and avoid overlaps.

Other partnerships have been established with the secondary healthcare system, particularly with several hospitals that have ensured the support and the monitoring of children in pediatric subspecialties.

The articulation with higher education institutions, which ensures the program’s theoretical and scientific support, was promoted, as well as with the Schools of Education of Portalegre, Beja and the Department of Psychology of Évora University. These institutions organize training and research activities on a regular basis.

At a local level, working groups promote combined efforts to ensure several services that ensure answers to families’ needs. At present, the program counts on the collaboration of 217 services: local authorities, health centers, schools, local social action services, other Social Solidarity institutions, security forces, volunteer firefighters, commissions for the protection of children, local development associations, amongst others.

Health services, especially family doctors, play an important role by signaling problematic situations and providing quick referral. Many early intervention teams are based in the health centers and this facilitates the articulation and the interconnection between the services and promotes early detection of developmental disorders.

All this networking increases the ability to provide solutions for complex problems inside the community, problems that go beyond the sectorial fields of education, health and social action, but which require integrated approaches/responses.

### Network Impact: Children, Families and Community

The theoretical framework that supports the early intervention network emphasizes that it can obtain several types of results. At the development level, it aims to optimize the use of brain plasticity and the children’s developmental potential, reducing the secondary effects of chronic disease and other disabilities. It promotes and reinforces attachment as a determinant factor in development, minimizes the negative influence of risk factors, and strengthens as well as enhances the child’s ability to live in society. This prepares the children for the entry and progression in the education system from the age of 6.

For the family, it reinforces appropriate patterns of interaction and parental skills, helping to reduce family stress brought on by the situation. It also allows for the strengthening of formal and informal networks of social support for the family and can help reduce elements of social exclusion.

At the community level, it also plays an important role by promoting the articulation between services and bringing them closer to the target population, increasing quality and making resources more cost effective. Finally, it promotes social inclusion, because the different partners are focused on problem-solving.

A study carried out to evaluate the effective impact of the Early Intervention network in the Alentejo (Franco and Apolónio, 2008) has equally shown the effectiveness of its accomplishments:

(A) There is an effective strengthening of the role of health centers in the detection, signaling and referral of cases and an increase in the skills of health professionals in the assessment of development disorders. The impact on the detection and referral of cases to LIT and on the beginning of the intervention by those professionals is especially important as it has significantly reduced the age of diagnosis and the onset of the intervention.

(B) Early Intervention teams are an important element of the families’ social support networks, being in many cases considered the most important element.

(C) Creation of the early intervention network had a strong impact on the activity of systems and services resulted in modifications in the practices of its professionals. With the increase of family centered work and a transdisciplinary functioning, professionals identified a strong impact on activity and services systems and a change in their own practices.

(D) Families found out a positive impact on children’s development and on being more responsive to their needs.

The Early Intervention program in Alentejo, as part of the SNIPI, can be an example of good practices, with its own characteristics that allowed the creation of a regional network of integrated and comprehensive responses to the needs of the population. Its innovative character has been recognized, nationally and internationally, by being awarded two prizes. In October 2009, the program won the 2nd Prize of Good Health Practices in the context of their participation in the “Award for good practices on fairness, effectiveness and efficiency in health,” awarded by APDH/HOPE & FIH, with Novartis Oncology support. In 2010, this same program was distinguished by WHO – World Health Organization with the United Arab Emirates Foundation Prize for Health, for the contribution to the development of health in Portugal. These distinctions reinforced the importance of the work already done and the necessary commitment to reinforce the positive aspects and overcoming constraints that still exist, in order to expand this model across the country.

## Conclusion

By looking at how children who receive early intervention find themselves in a better position to face the challenges of schooling and by evaluating the impact of the early intervention network at the level of the educational system, we confirm that:

(a) Children with developmental or educational problems need to receive support from a team of early intervention, with an effective network coverage;

(b) Kindergarten teachers who integrate these teams are experienced professionals. Most of them possess a specialized course, and all of whom have some experience in the field of early intervention (Dunst, 2009). They also consider themselves to be well-informed about early intervention (its theoretical foundations as well as its practices) and believe they have think they have increased their knowledge about development problems;

(c) Most of the teachers working in kindergarten classrooms do not possess any training in early intervention. However, their level of information about the early intervention system is high: they know how the network works, how to contact the LIT, and have in fact an effective contact with it and they know how to use the referral process;

(d) Each teacher works with a considerable number of children and families (about 10), and most of the children are aged from 3- to 5-years-old. There seems to be a good margin of progression in early intervention, and the number of children under 3 years is increasing;

(e) Children go to early intervention mainly as a result of being at risk situations because of family and social factors. Disability cases, or cases of severe developmental delays cases are significantly less. It is therefore important to:

- Define coherent methods to establish diagnosis or characterization of situations;

- Make a more specific characterization of children;

- Differentiate developmental aspects, for example, language skills, that requires working at the educational level from disabilities or to situations where pathology is present (Franco and Apolónio, 2011).

(f) The transition to elementary school is a very sensitive aspect, and includes the monitoring of other entities responsible for the protection of the child as well as for the social support to the family;

(g) Teachers recognize their practices reflect changes due to the work within the Early Intervention System. The bigger the use of specific assessment tools and intervention planning strategies, the more relevant change is brought on as result, empowering teachers ability to assess the needs of the child and the family and to promote changes in their own contexts. More than 80% of interventions are made at the child’s or in the kindergarten/nursery, twice a week or once a week (with a duration of over an hour each).

(h) The intervention of the teachers is no longer focused only on the child, but also on their parents, on other teachers inside the kindergarten and even on other professionals the child works with. The family-centered intervention is considered one of the most significant benefits brought to the practice of these early intervention teachers, such as the ability to recognize the strengths and capabilities of the child and his family. Their work with families is mainly providing information, referrals and counseling, in the context of a close relationship. The transdisciplinary perspective is enhanced and hones skills such as the ability to spot difficulties shown by the children.

A global network operation contributes to:

(a) Inclusion: facilitating the transition, based on a non-discriminatory approach and that repudiates exclusion. On the contrary, the child receives care and support in a specific setting and the family assumes the center stage in this interventions.

(b) Educational achievement: by strengthening the development, and an adapted contextualization in pre-school education. That can also be possible because the children go to school with an effective screen of their developmental needs as well as the level of support necessary for each of them.

Consequently the system introduces major innovation within the framework of educational policies that promote development and inclusion, which has deserved the interest of the scientific community and of policy makers. It has been possible to implement some of principles already studied but it had never been tested at this level. It must be said that, for their implementation they require a proper educational policy context.

## Author Contributions

VF: Conception and design, manuscript preparation, and final approval of the version to be published. MM: Contributions to the conception of the work and drafting of the work. GS: Contributions to the design of the work, critical revision, and final approval of the version to be published. AA: Study design and data collection. LA: Study design, data collection, and final revision.

## Conflict of Interest Statement

The handling Editor declared a shared affiliation, though no other collaboration, with several of the authors VF, MM, GS, and states that the process nevertheless met the standards of a fair and objective review. The other authors declare that the research was conducted in the absence of any commercial or financial relationships that could be construed as a potential conflict of interest.
